# Community Health Workers in School Systems: Social Prescribing for Healthcare Access and Resource Allocation

**DOI:** 10.3390/healthcare14091217

**Published:** 2026-05-01

**Authors:** Marcie Johnson, Kendra Summers, LaShawn McClary, Mindi B. Levin, Catherine Ling, Natalie Exum, Kimberly Hailey-Fair, Elisabeth Vanderpool, Rebecca Chen, Anthony Rivetti, Ursula E. Gately, Amanda Toohey, Jacqueline Bryan, Jordyn Gunville-Pourier, Z. Thomasina Watts, Meghan Brown, Olivia Banks, Brittany Martin, Annette Anderson, Panagis Galiatsatos

**Affiliations:** 1 Medicine for the Greater Good, Johns Hopkins University School of Medicine, Baltimore, MD 21224, USAkhailey1@jhmi.edu (K.H.-F.); evande16@jh.edu (E.V.); ugately1@jh.edu (U.E.G.); atoohey2@jhu.edu (A.T.); jgunvil1@jh.edu (J.G.-P.); pgaliat1@jhmi.edu (P.G.); 2 Department of Health, Behavior and Society, Johns Hopkins University Bloomberg School of Public Health, Baltimore, MD 21205, USA; mlevin@jhu.edu (M.B.L.); zwatts1@jhmi.edu (Z.T.W.); 3 SOURCE, The Community Engagement and Service-Learning Center, Johns Hopkins University Schools of Public Health, Nursing and Medicine, Baltimore, MD 21205, USA; 4 Department of Nursing Faculty, Johns Hopkins University School of Nursing, Baltimore, MD 21205, USA; cling11@jhu.edu; 5 Department of Environmental Health and Engineering, Johns Hopkins Bloomberg School of Public Health, Baltimore, MD 21205, USA; nexum1@jhu.edu; 6 Office of Civic Engagement and Opportunity, Johns Hopkins University, Baltimore, MD 21205, USA; jbryan8@jhu.edu (J.B.); mbrow279@jhu.edu (M.B.); obanks3@jhmi.edu (O.B.); bmarti74@jhu.edu (B.M.); 7 Johns Hopkins Center for Indigenous Health, Washington, DC 21231, USA; 8 Department of Counseling and Educational Studies, Johns Hopkins School of Education, Baltimore, MD 21218, USA; annette.anderson@jhu.edu; 9 Division of Pulmonary and Critical Care Medicine, Department of Medicine, Johns Hopkins University School of Medicine, Baltimore, MD 21224, USA

**Keywords:** health equity, community engagement, COVID-19

## Abstract

**Background:** During the early years of the coronavirus disease 2019 (COVID-19) pandemic, many schools found their staff, specifically teachers, adapting their roles to address social and health challenges, such as food insecurity and health literacy. Given the challenges these school-based communities faced, and continue to face, a clear gap was exposed during these early years of the public health crisis: a lack of community-centered professionals who can assist with social health factors impacting health and well-being. **Methods:** In this descriptive report, we examine the process and implementation of training two teachers to become community-centered professionals, specifically community health workers (CHWs), to serve schools located in socioeconomically challenged neighborhoods of urban regions. We explore their training and how these CHW–teachers prescribed social health interventions across four major domains: (a) access to medical and environmental equipment, (b) mental health challenges, (c) food insecurity, and (d) health literacy. We describe the specific interventions they implemented and the potential economic value and practicality of the overall initiative. **Outputs:** In less than one year, two teachers were successfully trained as CHWs in and for underserved communities. These CHW–teachers conducted informal surveys based on objectives of health themes that aligned with absenteeism. Both the process and implementation of the CHW training and CHW-led school-based interventions proved cost-effective and practical. **Conclusions:** Having CHW–teachers in schools is practical, may offer economic value, and is likely to complement additional health initiatives at schools (e.g., school nurses). As a small-scale pilot initiative, further studies should evaluate CHW–teacher impact on school-based goals, such as attendance rates, while this report focuses on early implementation processes.

## 1. Introduction

In the early years of the global COVID-19 crisis, numerous state governments in the United States closed schools in an effort to mitigate the spread of the infectious virus, SARS-CoV-2. For many schools, these closures in the spring of 2020 transitioned learning to virtual settings, in-person learning with infectious control policies in place, or a hybrid version of the two to maintain educational objectives while attenuating the spread of SARS-CoV-2 [1,2,3]. For those with in-person or hybrid learning formats, significant attention was placed on COVID-19 precautions, from face masking to social distancing and hand hygiene [2,4]. While COVID-19 precautions were prioritized, social challenges that existed before the pandemic re-emerged, and some were amplified during these early years. For many schools and their respective students, this forced limited resources to be stretched to tackle public health crises and regional social issues simultaneously [5]. These strains and challenges were placed upon school-based communities and their respective educators, faculty and staff with few options for relief or assistance.

However, community-based solutions to various public health concerns are often feasible and effective with the assistance of community brokers able to create and maintain weak ties (a sociological term conveying connections with persons and entities outside of one’s own social network) with various social organizations [6,7]. An example of a weak tie is a faith-based organization working with a healthcare system. For instance, the faith-based organization recognizes that there is a significant diabetes burden among their congregation and establishes a relationship with a healthcare system to provide a support group. The congregation identified a challenge (diabetes burden), recognized that solving that challenge is not within their organization’s expertise (known as structural holes), and established a relationship outside of their network (weak ties) with a healthcare system to assist. The maintenance of this relationship is effective through a liaison (community broker).

One type of a community broker that has gained attention over the last decade is the community health worker (CHW). A CHW is a public health worker who (a) lives in and/or is trusted by the community and (b) connects people to health and social services by breaking down barriers related to the social determinants of health [8]. CHWs, who are often employed by healthcare systems and public health departments and then deployed into communities, have been used extensively in population health strategies that target various public health concerns and medical issues [9,10,11].

In schools, when in-person learning resumed following the pandemic, educators continued to simultaneously navigate infectious disease mitigation and the persistent needs of students and their families, often without standardized guidelines that could be equitably applied across diverse regions or districts [12]. These conditions placed additional strain on schools already operating with limited resources. Given these pressures, integrating community-centered professionals into school settings emerged as one practical strategy to bridge structural gaps. The CHW–teacher pilot was initiated in this post-pandemic context to address these ongoing strains, providing structured support to teachers and schools still managing heightened social and health-related needs, even years following the pandemic.

In the initiative described here, existing teachers from two socioeconomically challenged urban neighborhoods were trained to become community-centered brokers—specifically, CHWs. While CHWs working within school systems is not a new concept [13], training teachers to function in a CHW-informed role represents a novel approach. In this model, a CHW–teacher is defined as a teacher who receives training in the foundational principles and skills of community health work to better identify social needs among students and families and connect them with appropriate resources. The aim was to help teachers learn to identify social needs, determine which challenges could be addressed within the school, and facilitate connections to external organizations when broader support was required. The model builds on the shared role of teachers and CHWs in supporting community well-being within school environments. Instead of providing direct service, the CHW–teacher role functions as a resource navigation and capacity-building model, strengthening teachers’ awareness of the social determinants of health and their ability to facilitate connections to school and community supports. This model does not replace school-based roles such as nurses, counselors, or social workers, who provide direct services. Rather, it provides an educational framework that strengthens teachers’ understanding of the social challenges students may experience outside of school and how these experiences can affect learning and classroom engagement. The current article serves as a program description, illustrating how teachers were trained to become CHWs and what school-based interventions they implemented following their training. The cost-effectiveness and practicality of this initiative are also highlighted.

## 2. Methods

This report follows an implementation-focused approach intended to describe the operational steps required to introduce the CHW–teacher model within school systems. While the CHW training program described below is a formalized, accredited training program, the school-based needs assessment and development and implementation of the school-based health engagement portions of the current program were implemented using informal, subjective processes that allowed for flexible, real-time adaptation and implementation. One aim of the future iterations of this program is to standardize these processes.

### 2.1. School and Neighborhood Demographic Variables

Two teachers were selected for this inaugural training cohort. Both teachers’ schools were located in socioeconomically disadvantaged neighborhoods of Baltimore City, as designated by their respective area deprivation index [14], known as the ADI (overseen by the University of Wisconsin School of Medicine, with funding by the National Institute on Aging), which is a composite score that the United States’ census tracts that measures the affluence (lower scores) to disadvantaged (higher scores) socioeconomic status of these regions [14]. The two ADIs for these schools were 86 and 91 out of 100. Both schools were elementary to middle school levels, with English as a Second Language (ESL) programs in place.

### 2.2. Community Health Worker Program

**Figure 1:**
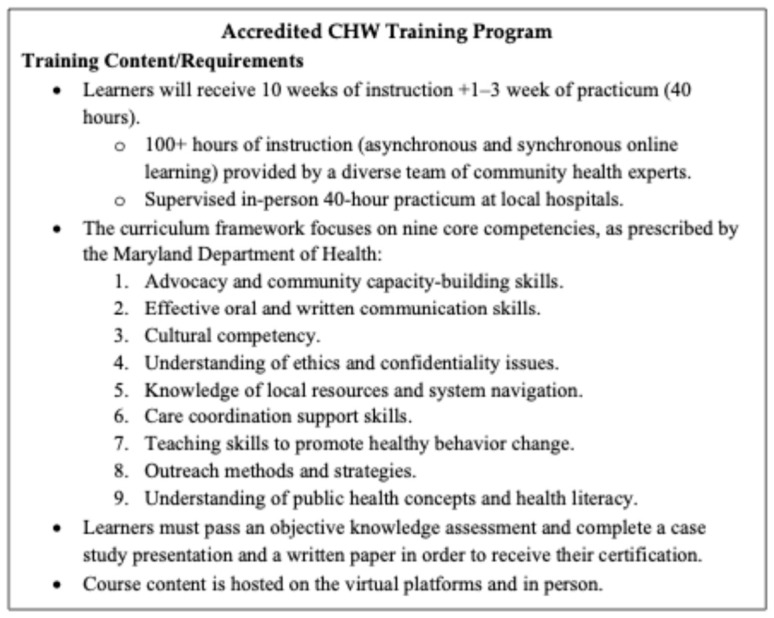
Community health worker training program overview.

Both educators enrolled in the Fall of 2024 into an accredited community health worker training program (accredited by the state of Maryland [15] and overseen by the Johns Hopkins Health System). The program is 10 weeks long with over 100 h of instruction, taught as a hybrid model (in-person and virtual learning), and is accompanied by a 40 h in-person practicum. The participating teachers enrolled voluntarily, with the tuition covered through a grant program, and their continued work following the training was supported through a state-sponsored apprenticeship and employment that provided stipends and salary support. Figure 1 provides a comprehensive list of all themes and the training timeline for CHW enrollees.

The teachers were recommended by the CHW training program lead after meeting with them to discuss their current roles within their schools and their interest in strengthening support for students and families. Both teachers had independently enrolled in the CHW training program to expand their knowledge of community resources and resource navigation. One teacher served as a community school coordinator, while the other was a health teacher at a different school. During initial discussions with the training program, both teachers expressed an interest in developing skills related to resource navigation, family engagement, and advocacy. To support the application of these skills, the teachers also participated in an apprenticeship component that provided additional on-the-job learning opportunities and educational support.

### 2.3. Implementation

**Figure 2:**
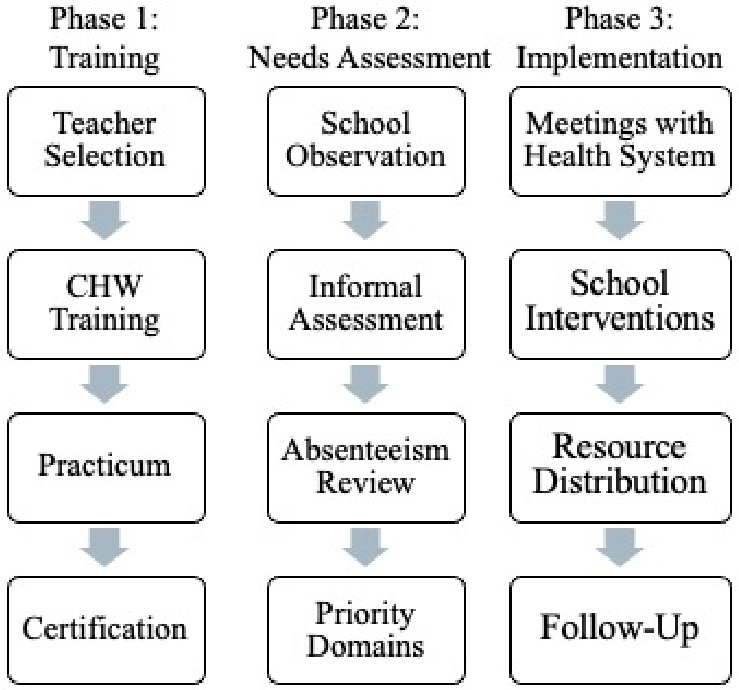
The implementation process for the CHW–teacher model. Note: A flowchart depicting the three-phase implementation process of the CHW–teacher model: Phase 1 (CHW training), Phase 2 (school and student needs assessments), and Phase 3 (implementation of targeted interventions).

To improve the transparency and reproducibility of the implementation process, the CHW–teacher initiative followed a structured three-phase workflow consisting of: (1) CHW training and certification, (2) school-based needs assessment, and (3) the development and implementation of school-based health engagements in partnership with healthcare system liaisons and community organizations (see Figure 2 for a visual summary of this process). Each phase included key activities: Phase 1 focused on CHW training and certification through the State of Maryland Health Department, Phase 2 involved identifying school health needs through informal surveys and observations, and Phase 3 included planning and implementing school-based interventions.

As detailed above, the CHW–teacher training was conducted through a Maryland accredited CHW training program. Following the completion of their CHW training, the teachers conducted informal assessments to identify health issues currently impacting their school communities. These informal assessments were conducted via written surveys or verbally, with real-time information collected by the teachers and aligned with previously identified school needs around reducing absenteeism. Other identified health issues were based on emerging school needs (e.g., a hand–foot–mouth outbreak at both schools). These informal assessments established the groundwork for future organized interventions with more formal data collection efforts for the school community.

Findings from CHW–teacher observations and surveys were then used to inform collaborative planning with healthcare system partners and community organizations, resulting in school-based interventions, resource distribution, and follow-up. Implementation outputs explored here include areas of need identified by the CHW–teachers, teacher-led engagements, the frequency and duration of engagements, and outputs and resources distributed during engagements. These outputs were tracked and recorded by healthcare system liaisons.

### 2.4. Cost and Planning

To document the practicality of implementing CHW–teachers in school settings, we assessed the financial resources and time requirements necessary to support program activities. This included examining the level of coordination required between school personnel and healthcare system partners and the financial resources needed to support training and program costs.

## 3. Results

### 3.1. Communities

These two CHW–teachers worked in two urban schools of Baltimore City, both socioeconomically disadvantaged (as previously mentioned, the respective ADIs were 86 and 91). School A serves 756 students from kindergarten to eighth grade, with 13% of students proficient in math and 22% proficient in reading. School B serves 694 students from kindergarten to eighth grade, with 5% of the students proficient in math and 21% proficient in reading.

### 3.2. Community Health Worker Program

Both teachers completed the training within the allotted 10-week timeframe and completed the 40 h practicum within the 3-week timeframe. The training was completed outside of the teachers’ work hours.

### 3.3. Implementation

Based on their observations, CHW–teachers identified four primary areas of health-related concerns in their communities: (a) access to medical and environmental equipment, (b) mental health challenges, (c) food insecurity, and (d) health literacy. These four domains of concern represented subjective community needs. The CHW–teachers also identified asthma and head lice as the leading causes of absenteeism in their students. While formal measures of healthcare access or attendance were not evaluated in this pilot, these resource navigation activities represent early indicators of how the CHW–teacher model may support improved access to care.

Using this information, the CHW–teachers, in partnership with the healthcare system liaisons, developed multiple school-based interventions that took place over the course of ten months. After CHW–teachers identified priority social and community needs within their school communities, volunteers of medical and undergraduate students who participate in the organization Medicine for the Greater Good provided logistical support for certain school-based activities. These volunteers assisted with tasks such as distributing materials, supporting outreach efforts, and helping facilitate program logistics during events. The CHW–teachers remained responsible for identifying needs, coordinating programming, providing access to families and school spaces, leading sessions, and conducting follow-ups with students and families. Volunteers supported implementation but did not independently determine program priorities. Table 1, Table 2, Table 3 and Table 4 summarize the CHW–teachers’ coordinated intervention activities by domain.

All school-based engagements were led by CHW–teachers, with trained healthcare system volunteers supporting logistics and material distribution. Across the four primary domains (access to medical and environmental equipment, mental health challenges, food insecurity, and health literacy) CHW–teachers coordinated activities, facilitated sessions with students and families, and provided follow-up support. The distributed resources included trash removal, air purifiers, greenhouses, inhaler spacers, lice kits, educational pamphlets, in-person parent and student information sessions, and food pantry and meal program access.

### 3.4. Cost and Planning

This entire initiative, including teacher CHW training and the interventions described, took place over the course of 10 months. The curricula implemented during the school year required seven planning meetings between CHW–teachers and healthcare system staff (one hour each) to ensure proper timing and maximum student engagement. Finally, the CHW training cost $1500 per trainee.

Regarding medical equipment allocation, the CHW–teacher partnerships with the healthcare system, along with the broader CHW network, resulted in no costs to the schools for items such as head lice treatments, asthma medication spacers, air purifiers, trash pickers and protective equipment. 

The CHW–teachers’ time and effort supporting food security, mental health, and health literacy activities also represents an important resource. The CHW–teachers voluntarily enrolled in the program based on their interest in supporting students’ social and health needs. They were recommended to participate in a state-sponsored apprenticeship program, which provided additional financial support, education incentives, and structured opportunities to engage with volunteers and health system stakeholders. Volunteers assisted with logistics, material distribution, and outreach, helping to offset some of the resource demands. Together, these supports enabled the teachers to implement program activities, and the initiative tracked outputs.

### 3.5. CHW–Teacher Perspectives

During 30 min weekly check-ins with the research staff, CHW–teachers also reflected on the feasibility and sustainability of the CHW–teacher model. Both teachers described the training, cost-of-living stipend, and apprenticeship support as important factors in making additional responsibilities manageable alongside their existing roles. They also noted that engaging families and balancing dual responsibilities required ongoing effort. Related to long-term sustainability, both teachers identified district-level involvement as a potential pathway for standardization, while also noting that such support would need more evidence demonstrating the overall value and impact of the CHW–teacher model over time.

## 4. Discussion

This descriptive report illustrates how two teachers in socioeconomically disadvantaged urban schools were trained as CHWs and subsequently implemented a series of school-based health engagements across four domains: access to equipment, mental health education, food insecurity support, and health literacy. The partnership between CHW–teachers, existing CHW networks, and the healthcare system enabled the delivery of targeted health programming and resource allocation without creating additional financial burdens for schools. The initiative also demonstrated that existing community networks can support the cost-efficient and practical implementation of health-related activities within school settings.

While the findings demonstrate that CHW–teachers can effectively leverage community and healthcare system partnerships to address health-related needs within school settings, an important consideration is the additional workload placed on educators who assume this dual role. Interviews with participating teachers highlighted that the feasibility of the CHW–teacher model is closely tied to role alignment, institutional support, compensation, and the individual fit of the teacher assuming the role.

For one participant whose existing position already included community coordination and family engagement, many CHW-related activities overlapped with established responsibilities, such as connecting families with external resources and serving as a liaison between the school and community partners. In this context, the CHW certification did not represent a fundamentally new workload but instead enhanced the effectiveness of existing duties by providing structured training, a formalized framework, and access to a broader network of supports. This alignment reduced role strain and allowed CHW activities to be integrated into daily work without substantial disruption. It also enabled more structural engagement with families and community partners, supporting health-related goals in a systematic way.

For the classroom-based teacher, CHW responsibilities were additive and required the intentional management of time. CHW-related tasks were typically completed during planning periods, lunch breaks, or outside of instructional hours. Prior teaching experience facilitated this dual role, as familiarity with curriculum planning and classroom demands reduced the burden of incorporating accessible, health-related content into existing lessons. Importantly, both participants noted that not all teachers would be well suited to this model. They emphasized the importance of identifying educators who are intrinsically motivated, willing to “go the extra mile,” and comfortable navigating responsibilities beyond traditional instructional roles. This insight suggests that the CHW–teacher model may be best implemented through intentional selection rather than broad adoption.

Compensation emerged as another critical factor in sustaining teacher engagement. Both participants described the stipend provided through the current pilot as generous and appropriately reflective of the additional time and effort required for CHW work and noted that this financial recognition made the expanded role worthwhile. This finding underscores that expecting teachers to assume additional responsibilities without compensation may limit participation and contribute to burnout, whereas stipends or supplemental pay structures can support feasibility and retention.

Participants also reflected on the broader question of sustainability and institutionalization. While both teachers identified district-level involvement as a potential long-term pathway, they acknowledged that such support would likely require evidence demonstrating the value and impact of CHW–teachers over time. These perspectives highlight key considerations for feasibility, workloads, and sustainability in implementing this pilot initiative. From their perspective, expecting immediate district adoption without established evidence of impact would be unrealistic. Instead, they identified alternative funding mechanisms—such as philanthropic support, partnerships with community-based organizations, investment from healthcare systems, or partnerships with local government—as more feasible short-term strategies to sustain and expand CHW–teacher initiatives.

Together, these perspectives suggest that successful implementation of the CHW–teacher model depends not only on structural supports and funding but also on careful role selection and phased approaches to sustainability. Overall, observations from this work highlight the potential value of integrating CHW-trained teachers into school communities as a strategy to address health-related needs that intersect with academic, behavioral, and social outputs. However, while promising, these results reflect a small-scale, early-stage implementation, and generalizability is limited. Because this pilot involved two teachers working within identifiable school communities, formal school-level outcome indicators such as attendance or healthcare utilization were not analyzed.

Importantly, although formal outcome measures were not systematically collected in this pilot, preliminary observations from participating schools suggest potential impact. One CHW–teacher reported that their school experienced an approximate 18% reduction in absenteeism during the year in which the CHW–teacher model was implemented. While this observation is descriptive, based on school-level reporting, and cannot be directly attributed to the intervention given the absence of a controlled design, it provides a potential proxy indicator of the model’s influence on student engagement and health-related barriers to attendance. In school systems where absenteeism is often driven by modifiable social and health factors, such as asthma, environmental exposures, and access to basic resources, improvements in attendance may reflect enhanced access to support and care. In addition, because school funding in many U.S. systems is partially tied to attendance, reductions in absenteeism may also suggest potential downstream economic implications, though formal cost analyses were beyond the scope of this pilot. These early observations highlight the importance of incorporating aggregated and anonymized outcome measures in future studies to more rigorously evaluate the impact of CHW–teacher initiatives. Future work should examine the longer-term impact of CHW–teacher activities on school-level indicators—such as attendance, health literacy, and family engagement—and assess the model’s applicability in diverse educational contexts, including rural and linguistically diverse communities. Additional research is also warranted to evaluate scalable funding mechanisms and strategies to support sustainability.

## 5. Conclusions

This study provides an illustrative example of how training teachers as community health workers can expand school capacity to address health-related needs in under-resourced communities. The findings suggest that the CHW–teacher model is both practical and resource-efficient, offering a structured approach to integrating social and health supports within educational settings.

Beyond this pilot, the model highlights the potential for schools to serve as critical access points for addressing social determinants of health through existing, trusted personnel. As additional research is conducted, future studies should evaluate the impact of CHW–teacher initiatives on measurable outcomes such as attendance, health access, and student well-being, as well as explore scalable funding and implementation strategies across diverse educational contexts.

## Figures and Tables

**Table 1:** Access to medical and environmental equipment engagement activities.

Domain	School	Engagement/Activity	Frequency and Duration	Outputs/Resources Distributed
Environmental health and equipment access	School A	Watershed restoration activity with live presentation on clean waterways; Grades 6–8	One 10 min presentation and hands-on restoration activity	Trash removal around local tributary
		Climate change curriculum culminating in air purifier construction; Grades 6–7	Eight sessions; 50 min each	Forty-two air purifiers constructed and distributed (box fans with MERV 10–13 filters)
		Greenhouse acquisition for future curriculum on food and climate	Not applicable	Greenhouse secured for the 2025–2026 school year
Indoor air quality and asthma education	School B	Anti-smoking and anti-vaping curriculum focused on indoor air quality; Grades 6–7	Six lessons; 50 min each	Provided instruction on indoor air quality risks
		Parent education on asthma symptoms and inhaler use	Two sessions; 30 min each	Twenty-two inhaler spacers distributed
Head lice support	Both schools	Individual family outreach for head lice education and treatment distribution	As needed; individualized	Eighty-four lice kits distributed (40 at School A; 44 at School B)

Note: All engagements were implemented by CHW–teachers in partnership with trained healthcare system volunteers. The session length is reported in minutes. Grade levels reflect the U.S. educational system. MERV = Minimum Efficiency Reporting Value.

**Table 2:** Mental health engagement activities.

Domain	School	Engagement/Activity	Frequency and Duration	Outputs/Resources Delivered
Mental health education	Both schools	Mental health curriculum developed by mental health expert faculty; Grades 6–7	Four lessons; 50 min each	Provided information about mental health concepts and coping strategies
Trauma-informed support	School B	Trauma-informed community session conducted in response to a school incident	One session; duration based on community need	Provided trauma-informed support and education for community members

Note: All sessions were delivered by trained volunteers from the partnering healthcare system [16]. Grade levels reflect the U.S. educational system. The session length is reported in minutes.

**Table 3:** Food insecurity engagement activities.

Domain	School	Engagement/Activity	Frequency and Duration	Outputs/Resources Delivered
Food insecurity support	Both schools	Provision of food at parent–teacher morning and evening meetings	Monthly events throughout the academic year	Consistent food availability during school-based family events
	Both schools	Assistance with enrollment in meal support programs (e.g., Meals on Wheels)	As needed; individualized	Five families in total enrolled in long-term meal programs

Note: Food insecurity activities included both short-term (event-based food provision) and long-term (meal program enrollment) supports. Enrollment assistance was provided through the CHW–teachers’ community health worker network. The frequency reflects the standard academic calendar.

**Table 4:** Health literacy engagement activities.

Domain	School	Engagement/Activity	Frequency and Duration	Outputs/Resources Delivered
Health literacy promotion	Both schools	Integration of health literacy concepts across all curricula (environmental health, air quality, mental health) with pedagogy tailored to student needs	Embedded throughout all lessons; duration aligned with corresponding curricula	Supported student comprehension of health concepts through age-appropriate instruction
	Both schools	Meetings with families accompanied by invited health experts (topics included water quality, asthma, air quality, mental health, and trauma care)	Occurred during scheduled family meetings; duration varied	Supported family understanding of health topics; follow-up provided as requested

Note: Health literacy activities were embedded across all CHW–teacher programming. Pedagogy was tailored collaboratively with education faculty to ensure age-appropriate delivery. Health experts were invited to family meetings based on community-identified priorities, and CHW–teachers facilitated follow-up when needed.

## Data Availability

The data presented in this study are not publicly available due to the small sample size and associated risk of participant identification. The sharing of de-identified data is further restricted by ethical considerations and institutional review board (IRB) requirements. Data may be made available from the corresponding author upon reasonable request, subject to appropriate safeguards and approvals.
